# Highly Efficient Flame-Retardant and Enhanced PVA-Based Composite Aerogels through Interpenetrating Cross-Linking Networks

**DOI:** 10.3390/polym15030657

**Published:** 2023-01-27

**Authors:** Ningjing Wu, Shanshan Deng, Fei Wang, Mohan Wang, Mingfeng Xia, Hongli Cui, Haoyi Jia

**Affiliations:** Key Laboratory of Rubber-Plastics, Ministry of Education/Shandong Provincial Key Laboratory of Rubber-Plastics, Qingdao University of Science & Technology, Qingdao 266042, China

**Keywords:** poly(vinyl alcohol), composite aerogel, flame retardancy, silane, interpenetrating cross-linking

## Abstract

Poly(vinyl alcohol) (P)/alginate (A)/MMT (M) (PAM) composite aerogels was modified through interpenetrating cross-linking of methyltriethoxysilane (Ms) or γ-aminopropyltriethoxysilane (K) and calcium ion (Ca^2+^) as a cross-linking agent, respectively. The compressive moduli of the cross-linked PAM/MsCa and PAM/KCa aerogels greatly increased to 17.4 and 22.1 MPa, approximately 10.5- and 8.2-fold of that of PAM aerogel, respectively. The limited oxygen index (LOI) values for PAM/MsCa and PAM/KCa composite aerogels increased from 27.0% of PAM aerogel to 40.5% and 56.8%. Compared with non-cross-linked PAM aerogel, the peak heat release rate (PHRR) of PAM/MsCa and PAM/KCa composite aerogels dramatically decreased by 34% and 74%, respectively, whereas the PAM/KCa aerogel presented better flame retardancy and lower smoke toxicity than the PAM/MsCa aerogel because of the release of more inert gases and the barrier action of more compact char layer during the combustion. The highly efficient flame-retardant PAM-based composite aerogels with excellent mechanical properties are promising as a sustainable alternative to traditional petroleum-based foams.

## 1. Introduction

Aerogels are promising materials in a wide range of fields, including supercapacitor [1,2], electromagnetic wave absorption materials [3], oil-water separation materials [4,5], superabsorbent [6,7,8], catalyst support [9,10], and thermal insulation materials [11,12,13], because of their outstanding advantages including super high specific area, low density, high porosity, and low thermal conductivity [14,15,16]. So far, a variety of polymer-based aerogels, including poly(vinyl alcohol) [5,9,11,17], cellulose [11,18,19,20,21,22], alginate [23], chitosan [24], and pectin [25] have been developed. With the incorporation of polymer matrix, the inorganic/polymer composite aerogels exhibited lower pore size, higher density, lower thermal conductivity, and better mechanical properties, compared to those of inorganic aerogels [17,18,19,20,21,22,23,24,25]. However, the incorporation of flammable polymers could result in the decrease of the flame retardancy of such polymer/inorganic composite aerogels, which restrict their potential application in the thermal insulation materials. Therefore, it is necessary to develop high-performance polymer-based composite aerogels with superior fire safety, good mechanical properties, and good water resistance.

Various inorganic micro- and nano-fillers, such as montmorillonite [26], graphene [26,27,28], hydroxyapatite (HAP) [29], sodium bicarbonate [30], and layered double hydroxides [31], have been used to improve the flame retardancy of polymer aerogels. One used case is that the incorporation of HAPs into poly(vinyl alcohol) (PVA) led to a significant decrease in the peak heat release rate (pHRR) and total heat release (THR) of PVA aerogels. The PVA/HAP composite aerogels presented lower thermal conductivity and higher specific modulus than those of polystyrene (PS) and polyurethane (PU) foams, and the silane-modified PVA/HAP composite aerogels exhibited a self-cleaning capacity and water resistance, which made them promising candidates for building thermal-insulation materials [29].

Nowadays, the mechanical properties of polymer aerogels were enhanced by the incorporation of various cross-linking agents, such as aldehydes [32], divinylsulfone [33], cellulose nanofibrils [18], borax [34], CaCl_2_ [35], and bio-based gelatin [36]. For example, the incorporation of a bio-based gelatin cross-linker could simultaneously improve the mechanical properties and flame retardancy of PVA-based aerogel by tailoring the network microstructure [36]. The cross-linked clay/PVA aerogel with an aliphatic polyisocyanate and 3-glycidoxypropyl trimethoxysilane as cross-linkers showed significantly improved mechanical, dimensional stability, and moisture resistance [37]. The PVA composite aerogel exhibited ultrahigh compressive property, owing to the incorporation of a double chemically and physically cross-linked system between the PVA, phytic acid (PA), and MMT [38].

Methyltriethoxysilane (MTES) and γ-aminopropyltriethoxysilane (APTS) are both important silane coupling agents and the multi-hydroxyl formed by the hydrolysis of silane could produce cross-linking network with PVA hydrogel, in order to enhance the mechanical property of the aerogels. Silane as a coupling modifier of fillers can improve the interfacial compatibility between fillers and polymer matrix. However, a small amount of the literatures reported the influence of silanes with different structures as the cross-linking agents on the morphology and mechanical properties of aerogels. In this manuscript, a series of highly efficient flame-retardant PAM composites aerogels were fabricated by the incorporation of different silane and CaCl_2_ as a cross-linker, respectively. The influence of two kinds of silane cross-linking agents with different structures (such as MTMS and APTS) on the morphology and compressive properties of PVA-based composite aerogels were compared, and the flame-retardant performance and its flame-retardant mechanisms of the as-prepared cross-linked PVA-based composites were systematically investigated.

## 2. Experimental

### 2.1. Materials

Poly(vinyl alcohol) (PVA-1799, degree of polymerization: 1700, degree of hydrolysis is 99%) was supplied from Shanghai Epi Chemical Reagent Co., Ltd., Shanghai, China. Sodium alginate (A) was purchased from Tianjin Zhiyuan Reagent Co., Ltd., Tianjin, China. Montmorillonite (MMT) was produced from Shijiazhuang Chenxi Refractory Materials Co., Ltd., Shijiazhuang, China. Methyltriethoxysilane (MTMS or Ms) and γ-aminopropyl-triethoxysilane (APTS or K) were purchased from Zhejiang Chemical Technology Co., Ltd., Hangzhou, China. CaCl_2_ was purchased from Shanghai Sinopharm Chemical Reagent Co., Ltd., Shanghai, China.

### 2.2. Preparation of the Cross-Linked PAM Aerogels

A PAM composite hydrogel was prepared according to the previous literature, and the weight ratios of PVA, alginate, and MMT components were 4 wt%:4 wt%:4 wt% in deionized water, respectively [36]. The pH of the PAM hydrogel was adjusted to 11 by adding ammonia water, and 2 wt% silane was slowly dropwise added and stirred for 5 min at 1000 rmp, until the viscosities of dispersion obviously increased. The cross-linked PAM aerogels were obtained after the freeze-drying of the hydrogel in a lyophilizer at −50 °C and 10 Pa for 48 h. The prepared sample was referred as PAM/Ms or PAM/K aerogel, where MTES and APTS as the cross-linking agents referred to Ms and K, respectively.

**Scheme 1:**
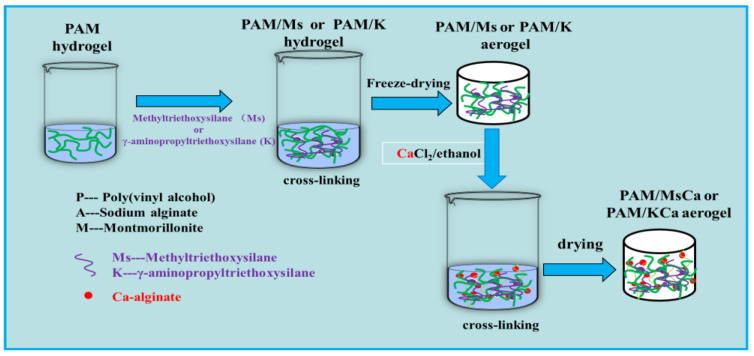
Preparation process of the different cross-linked PAM composte aerogels.

The PAM/Ms or PAM/K aerogel was further cross-linked in a CaCl_2_ saturated ethanol solution for 12 h at 20 °C, and then it was dried to remove ethanol in a vacuum oven at 60 °C for 12 h. The resulting cross-linked PAM aerogel were referred as PAM/MsCa and PAM/KCa aerogels, respectively. The preparation process of the different cross-linked PAM composite aerogels is presented in Scheme 1.

### 2.3. Characterization

The microstructure of the aerogels was firstly characterized by a Vertex 70 type Fourier-transform infrared (FT-IR) spectrometer (Billerica, MA, USA, Germany Brucker Company), using a KBr disk in the wavenumber range of 4000–400 cm^−1^, and the crystalline structure of the sample was characterized using a D/Max-2500 type X-ray diffraction (XRD) instrument in the 2θ range from 4° to 60° at room temperature. The thermal property of the sample was measured by a Q500 Thermogravimetric Analyzer (TA Instruments, New Castle, DE, USA) at 10 °C/min from 25 to 700 °C under N_2_ atmosphere. The morphology of the sample was observed by a SM-6700F scanning electron microscopy (SEM) (Japan Electronics Corp., Tokyo, Japan), and the surface elemental compositions of the sample were measured by an INCA type energy dispersive spectrometer (EDS).

Compressive properties of the cylindrical aerogel specimens (the size of sample: 2 cm in diameter and 2 cm in height) were measured using a Zwick Z020 Universal Testing Machine (Ulm, Germany), with a load of 5000 N and a compression rate of 10 mm/min.

Flame retardant performance of the aerogel samples (125 × 10 × 10 mm^3^) was measured to obtain a UL-94 vertical burning rating, according to ASTM D3801-2010 by a CZF-2 type instrument (Jiangning Analysis Instrument Co., Nanjing, China). The limited oxygen index (LOI) values of the aerogel samples (120 × 10 × 10 mm^3^) were measured according to ASTM D2863-2009 by a HC-2C type oxygen-index meter (Jiangning Analysis Instrument Co., Nanjing, China). Cone calorimeter test of the aerogel samples (100 × 10 × 10 mm^3^) was conducted according to ASTM E1354/ISO 5660 by a cone calorimeter (Suzhou Vouch Testing Technology Co., Ltd., Suzhou, China) at a heat flux of 35 kW/m^2^.

## 3. Results and Discussion

### 3.1. Microstructure of the Cross-Linked PAM Aerogels

**Figure 1:**
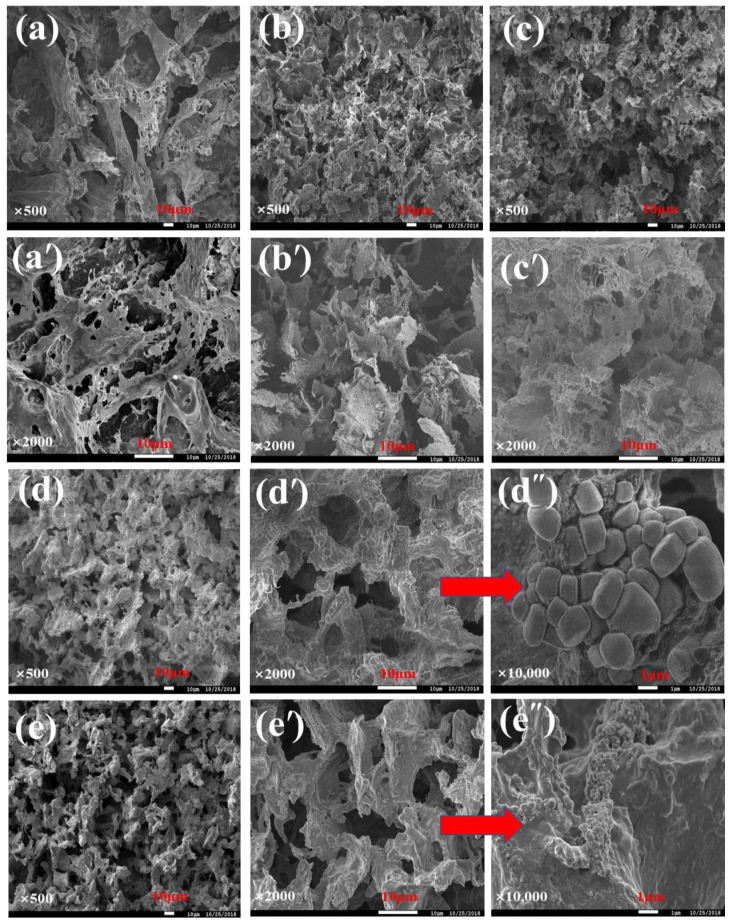
SEM images of the different PAM composite aerogels. (**a**,**a′**) PAM, (**b**,**b′**) PAM/Ms, (**c**,**c′**), PAM/K, (**d**,**d′**,**d″**) PAM/MsCa, (**e**,**e′**,**e″**) PAM/KCa.

The morphologies of the different PAM composite aerogels at the fractured surface by SEM measurement, as shown in Figure 1. The PAM aerogel exhibited a stacked cellular and unordered structure, and the cell size ranged from a few micrometers to dozens of microns (Figure 1a,a′), whereas the PAM/Ms and PAM/K composite aerogel presented more compact network bubble structures than that of non-cross-linked PAM aerogel (Figure 1b,c), and the different pore sizes were from dozens of nanometers and several microns (Figure 1b′,c′). It was attributed to that Si-OH groups of hydrolyzed MTMS or APTS in the PAM/Ms, and PAM/K hydrogels were reacted with –OH groups of alginates and PVA to form a three-dimensional cross-linked network structure. Additionally, the intermolecular hydrogen bonding interaction between amino (–NH_2_) groups of APTS with hydroxy(–OH) groups of PVA and SA in the PAM/K hydrogel was enhanced; thus, the size of pores for the PAM/K aerogel became smaller and, thus, the pore size of the PAM/K aerogel became smaller than that of PAM/Ms aerogel, and the pore distribution of the PAM/K aerogel was more uniform than that of PAM/M aerogel (Figure 1b′,c′).

After the step-by-step cross-linking of the PAM aerogel with silanes and of calcium ions, the bubble pore structures of the PAM/MsCa and PAM/KCa aerogels became more compact than those of the PAM aerogel (as shown in Figure 1d,d′,e,e′), respectively. It could be observed in Figure 1d′ that many in situ formed Ca-alginate particles were accumulated at the surface of bubble pore wall in the PAM/MsCa aerogel, and the size of particles were ranged from 1–2 μm. As shown in Figure 1e″, many Ca-alginate particles were embedded in the pore wall of PAM/KCa aerogels, and the particle size was less than 100 nm in the enlarged SEM images. The morphological difference of PAM/KCa and PAM/MsCa aerogels was attributed to stronger hydrogen-bond interaction between the amino groups of hydrolytic APTS and carboxyl groups (–COO^−^) of Ca-alginate particles of PAM/KCa aerogel than that of PAM/MsCa aerogel.

**Figure 2:**
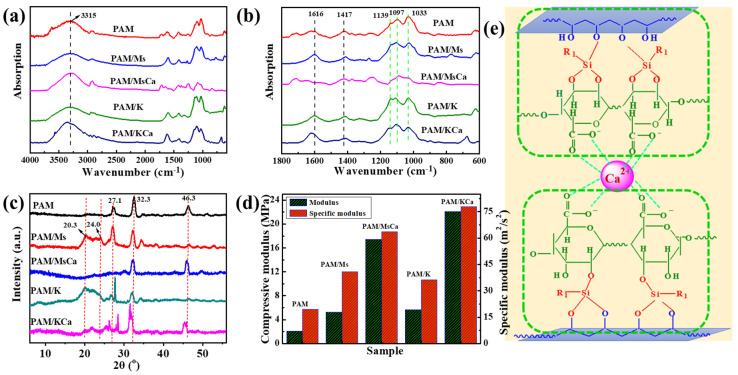
(**a**) FT-IR spectra and enlarged spectra of the different PAM composite aerogels in the range of 4000–600 cm^−1^, (**b**) 1800–600 cm^−1^; (**c**) XRD patterns of the different PAM composite aerogels; (**d**) Compressive properties of the different PAM composite aerogels. (**e**) Schematic diagram of interpenetrating cross-linking (IPN) network mechanism of PAM aerogels with silane and Ca-alginate.

In the FT-IR spectra of the different PAM composite aerogels (Figure 2a), the absorption peaks for the PAM aerogel around 3600–3000 cm^−1^ presented a very broad O–H stretching vibration band, and the characteristic peaks at 1616 and 1417 cm^−1^ were assigned to the asymmetric and symmetric stretching vibrations of carboxyl anions salt groups of alginates [35], respectively, and the peaks at 1033 and 1139 cm^−1^ were corresponding to the C–O–C stretching vibration from cyclic ether bridge of alginate, respectively. In the enlarged spectra of the PAM/Ms and PAM/K aerogels (as shown in Figure 2b), the characteristic peak at 1417 cm^−1^ of alginate shifted to 1413 cm^−1^, and the C–O stretching peaks at 1097 shifted to 1110 cm^−1^, whereas the peak intensity at 1139 cm^−1^ was enhanced, which was ascribed to the formation of new characteristic absorption peak of the Si–O–C through the hydroxyl condensation between hydrolyzed silane, PVA, and alginate. In the FT-IR spectra of the PAM/MsCa and PAM/KCa composite aerogels, the C–O peak intensities of aerogels at 1033 cm^−1^ obviously decreased with the incorporation of calcium ions, which was because the cross-linked “egg–box–like’’ microstructure of calcium alginate restrained the chain motion of the PAM/MsCa and PAM/KCa composite aerogels [36].

In the XRD patterns of the cross-linked PAM aerogels (Figure 2c), the diffraction peak intensity peak of alginate at 46.3° in the PAM/Ms and PAM/K aerogels was obviously weakened, whereas new characteristic diffraction peaks at 20.3, 22.1, and 24.1° appeared. This was attributed to the formation of a new cross-linked structure between the hydrolyzed condensation among the PAM/Ms and PAM/K aerogels.

For the PAM/MsCa and PAM/KCa composite aerogel, the diffraction peaks of alginate at 32.24° shifted to 32.20 and 31.40°, respectively, which were ascribed to the “egg–box” cross-linked structure between Ca^2+^ and carboxyl anions of alginates because the interpenetrating cross-linking structure of the PAM/MsCa and PAM/KCa composite aerogel had suppressed the chain movements. Thus, the new characteristic diffraction peaks of PAM/MsCa and PAM/KCa aerogel in the range of 20.0–25.0° was dramatically weakened. The interpenetrating cross-linking (IPN) network mechanism of the different PAM aerogels was illustrated in Figure 2e.

### 3.2. Mechanical Performance

The mechanical performance of the different PAM cross-linked aerogels is shown in Table 1. For the PAM/Ms and PAM/K aerogels, the porosities slightly decreased from 99.2% of PAM aerogel to 98.5% and 98.2%, respectively; however, their porosities remained above 95%. The densities of the PAM/MsCa and PAM/KCa composite aerogels obviously increased because a great deal of calcium alginate microparticles were embedded on the pore cell.

As shown in Figure 2d, the compressive moduli of the PAM/Ms and PAM/K composite aerogels increased to 5.3 and 5.7 MPa, 152% and 174% higher than that of the PAM aerogel, respectively, whereas the specific moduli of the PAM/Ms and PAM/K aerogels increased to 40.9 and 36.3 m^2^/s^2^, approximately 109% and 86% higher that of the PAM aerogel, respectively. It was because the MTMS or APTS as cross-linking agent enhanced the cross-linking densities of the PAM/Ms or PAM/K aerogel. The compressive moduli of the PAM/MsCa and PAM/KCa aerogels further increased to 17.4 and 22.1 MPa, 2.3- and 2.8-fold higher than those of the PAM/Ms and PAM/K aerogels, respectively. The specific moduli of the cross-linked PAM/MsCa and PAM/KCa aerogels also obviously increased, which was because in situ formed calcium alginate particles entered the three-dimensional network structure as physical cross-linking points at the bubble wall, resulting in an apparent improvement of the compressive properties of the PAM aerogels. In addition, the compressive modulus of the PAM/KCa aerogel was higher than that of the PAM/MsCa aerogel, which was attributed to that of the amino groups of APTS, which enhanced the hydrogen-bond interaction of the PAM/KCa aerogel.

### 3.3. Thermal Property

**Figure 3:**
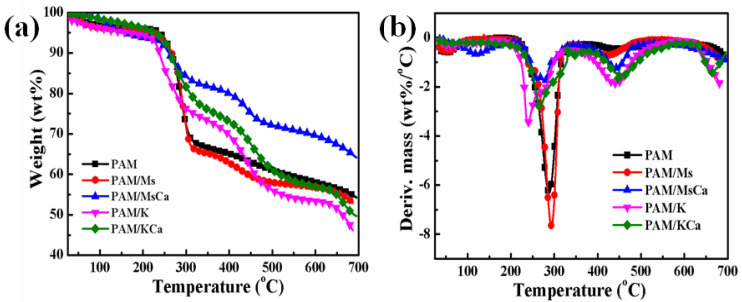
TG (**a**) and DTG curves (**b**) of the different PAM composite aerogels (N_2_ atmosphere).

The thermal decomposition process and thermal stability of the different PAM composite aerogels were investigated, and the corresponding thermogravimetric (TG) and differential thermogravimetric (DTG) curves are shown in Figure 3a,b, respectively. The thermal decomposition temperature at 5% weight loss (*T*_5%_) of the PAM/Ms aerogel decreased to 234.0 °C (Table 2), the first maximum thermal loss temperature (*T_max1_*) increased to 301.9 °C, and the corresponding maximum thermogravimetric loss rate (*dW/dt*) increased to 7.7%·min^−1^, which were mainly due to the dehydration reaction between PVA, SA, and hydroxylateds silane. For the PAM/K aerogel, the *T*_5%_ and *T_max1_* further reduced, and the second maximum *dW/dt* concomitantly increased, which were mainly attributed to the decomposition of amino- and alkyl groups of the cross-linked PAM/K aerogel, thus leading to the decrease of the residue yields of the PAM/Ms and PAM/K aerogels at 700 °C to 51.5% and 46.2%, respectively. For the PAM/MsCa and PAM/KCa aerogels, the *T_max1_* and *T_max2_* values were higher than those of the PAM/Ms and PAM/K aerogels, and the corresponding maximum weight loss rates decreased from 6.3%/min of the PAM aerogels to 1.8%/min and 2.9%/min; hence, the residue yields of the PAM/MsCa and PAM/KCa aerogels increased to 64.1% and 49.6%, respectively. The incorporation of Ca-alginate particles promoted the formation of the residues. Thus, the char yield of the PAM/MsCa and PAM/KCa aerogels were obviously higher than the corresponding PAM/Ms and PAM/K aerogels, respectively.

### 3.4. Flame Retardancy

**Figure 4:**
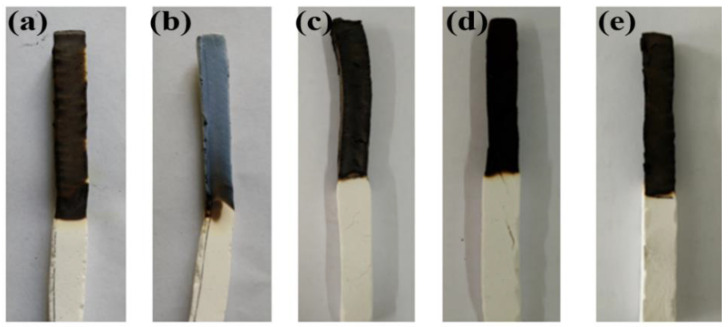
Digital photographs of the different PAM composite aerogels after LOI test. (**a**) PAM, (**b**) PAM/Ms, (**c**) PAM/MsCa, (**d**) PAM/K, (**e**) PAM/KCa.

The flame-retardant performance of the different PAM aerogels is listed in Table 3, and the digital photographs of the different PAM aerogels after LOI test are shown in Figure 4. The LOI of the PAM/Ms and PAM/K aerogels slightly decreased from 27.0% of the PAM aerogel to 26.8% and 26.5%, respectively, and their UL-94 rating remained V-0. This was mainly attributed to the fact that the alkyl groups of the cross-linked silanes were easily decomposed to produce combustible gases (such as low molecular alkanes, alkenes, and carbonyl compounds), which resulted in the slight decrease of the flame retardancy. The in situ formed calcium alginate particles in the cross-linked PAM aerogels performed as an intrinsic flame retardant, and a large quantity of Ca-alginate particles at the pore wall of PAM/MsCa and PAM/KCa aerogels were promoted to form compact char layer to inhibit the further combustion of inner aerogels, and the LOI values for PAM/MsCa and PAM/KCa aerogels dramatically increased to 40.5% and 56.8%, respectively. Therefore, the flame retardancy of the PAM/MsCa and PAM/KCa aerogels were dramatically enhanced.

### 3.5. Cone Calorimeter Test

**Figure 5:**
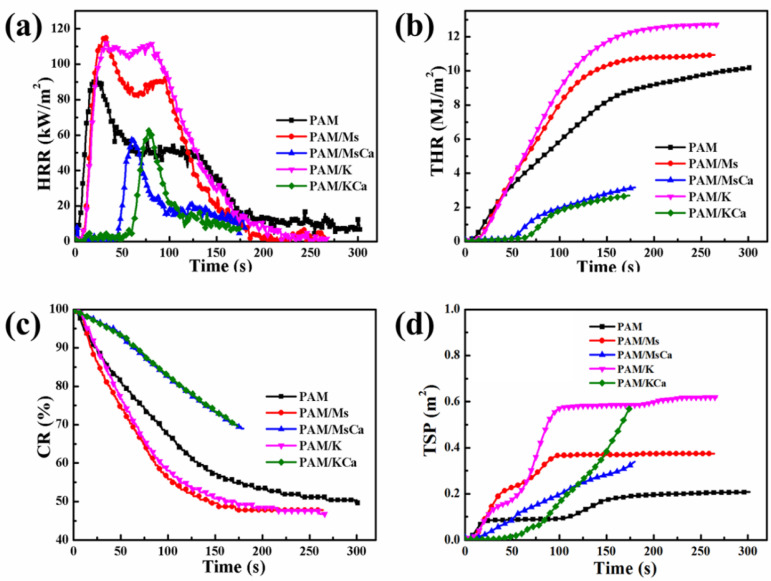
Cone calorimeter curves of the different PAM aerogels. Heat release rate (HRR) of the different PAM composite aerogels versus time curves (**a**), Total heat release (THR) versus time curves (**b**), Char residues (CR) yields versus time curves (**c**), and (**d**) Total smoke release (TSR) versus time curves at a heat flux of 35 kW/m^2^.

The cone calorimeter test data of the different PAM composite aerogels are compared in Table 3. The heat release rate curves (Figure 5a) of the PAM/Ms aerogel presented a double peak, and the first PHRR value increased to 114.8 kW/m^2^, whereas the first PHRR value of the PAM/K aerogel increased to 112.2 kW/m^2^. This was mainly attributed to that the silanes as cross-linking agents were decomposed to produce some flammable gases during combustion. Thus, the THR values (Figure 5b) of the PAM/Ms and PAM/K aerogels increased to 10.9 MJ/m^2^ and 12.7 MJ/m^2^, respectively, and the TSR values (Figure 5d) of the PAM/Ms and PAM/K aerogels increased to 41.1 m^2^/m^2^ and 64.5 m^2^/m^2^, respectively. In addition, the final CR yields (Figure 5c) of the PAM/Ms and PAM/K aerogels after cone calorimeter test decreased from 50.0% of the PAM aerogel to 47.7% and 46.8%, respectively. The result indicates the flame retardancy of the PAM/Ms and PAM/K aerogels slightly decreased by the cross-linking of silanes.

The HRR curves of the PAM/MsCa and PAM/KCa aerogels became single peak, and the corresponding PHRR values of the PAM/MsCa and PAM/KCa composite aerogels were dramatically decreased to 57.4 and 62.5 kW/m^2^, respectively. For the PAM/MCa and PAM/KCa aerogels, the THR values decreased to 3.2 and 2.7 MJ/m^2^, respectively. Meanwhile, the TSR values of PAM/MsCa and PAM/KCa composite aerogels significantly decreased to 8.6 and 5.5 m^2^/m^2^, and the final CR of PAM/MsCa and PAM/KCa dramatically increased to 69.0% and 69.6%, respectively. The result indicates that the flame retardancy of the PAM/MsCa and PAM/KCa composite aerogels was obviously improved and the smoke toxicity was greatly suppressed by two-step cross-linking.

The fire performance index (FPI) is the ratio of the ignition time (TTI) to PHRR. The TTI and FPI values of the PAM/Ms and PAM/K composite aerogels had no obvious change (Table 4), whereas the TTI of the PAM/MsCa and PAM/KCa composite aerogels were obviously extended to 44 s and 58 s, and the FPI value of PAM/MsCa and PAM/KCa composite aerogels increased to 0.77 and 0.93 s·m^2^·kW^−1^, respectively. Generally, the larger the FPI value is, the better the fire safety is. The results show that the fire safety of the PAM/MsCa and PAM/KCa aerogels was significantly improved by two-step cross-linking with silane and Ca^2+^.

THR/TML value (the heat release rate per gas mass) is a commonly used parameter to analyze the flame retardant mechanism in the gas phase, and the reduction of THR/THL value indicate there is a gaseous flame retardant mechanism in the flame-retardant polymer materials [39,40]. The THR/TML values for the PAM/Ms and PAM/K aerogels increased to 1.88 and 1.93 MJ·m^−2^·g^−1^, respectively, because the decomposition of combustible gases in the PAM/Ms and PAM/K aerogels in the gas phase resulted in the slight decrease in the flame retardancy. The THR/TML values of the PAM/MCa and PAM/KCa aerogels obviously decreased to 0.51 and 0.46 MJ·m^−2^·g^−1^, respectively, because the decomposed inert gases (such as H_2_O, CO_2_, etc.) of the PAM/MsCa and PAM/KCa aerogels in the gas phase were conductive to the improvement of flame retardancy.

### 3.6. Microstructure of Char Residue

**Figure 6:**
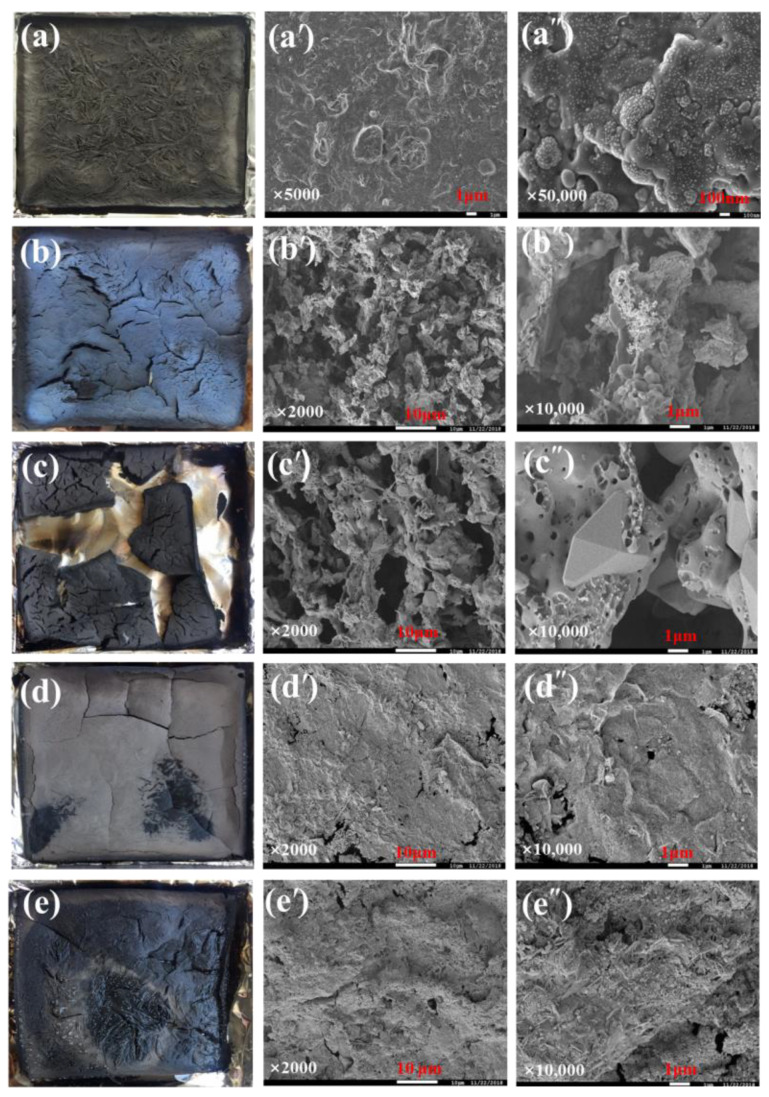
Digital photographs (**a**–**e**) and SEM (**a′**–**e′**,**a″**–**e″**) images of the residues for the PAM composite aerogels after cone calorimeter test. (**a**,**a′**,**a″**) PAM; (**b**,**b′**,**b″**) PAM/Ms; (**c**,**c′**,**c″**) PAM/MsCa; (**d**,**d′**,**d″**) PAM/K; (**e**,**e′**,**e″**) PAM/KCa.

The digital photographs and SEM images with different magnification of the char residues for the different PAM aerogels after cone calorimeter test could be compared in Figure 6. In the case of PAM aerogels, a continuous char layer structure was formed (Figure 6a), and a great deal of microparticles were scattered at the surface of the char (Figure 6a′,a″). The char morphology of the PAM/Ms aerogel (Figure 6b) exhibited a kind of relatively loose porous char structure with some obvious cracks and different scales of stacking particles embedded in the residue (Figure 6b′,b″). In the case of PAM/K composite aerogel, the char was split into a few pieces of irregular blocks (Figure 6c), which form some large pores of approximately 10 μm (Figure 6c′,c″). The porous microstructure of the char residues of the PAM/Ms and PAM/K aerogels could not effectively prevent the diffusion of the combustible gases and the heat transfer of into the interior layer; thus, the flame retardancy of the PMA/Ms and PMA/K aerogels decreased to a certain extent.

For the PAM/MsCa aerogel, the formed char residue was very compact (Figure 6d), and there were few bubbles in the char residue (Figure 6d′,d″). For the PAM/KCa aerogel, the formed char layer structure was much more continuous and compact (Figure 6e). There were many irregular and rod-shape nanoparticles, which were originated from the decomposition products (CaO or CaCO_3_) of calcium alginate (Figure 6e′,e″).

**Figure 7:**
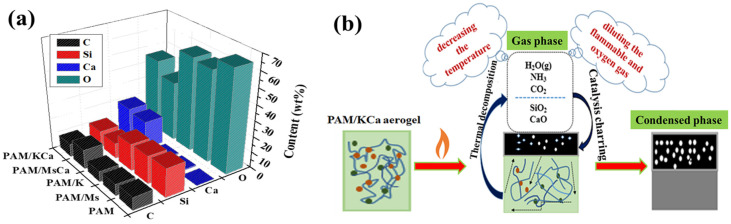
(**a**) Relative elemental compositions of the residues for the different PAM composite aerogels after cone calorimeter test by SEM–EDS measurement; (**b**) Schematic diagram of the possible flame retardancy mechanism of the PAM/KCa aerogel.

The average relative weight compositions of C and O elements in the PAM/Ms and PAM/K residues by EDS decreased, compared with those of the PAM aerogel (Figure 7a), which was mainly attributed to the thermal decomposition of the silicone as cross-linking agents. The compositions of Ca and O elements in the PAM/MsCa char increased to 12.4 wt% and 17.7 wt%, respectively. It indicates that the cross-linking of MTMS/Ca^2+^ in the PAM/MsCa aerogel promoted to the formation of the compact char residue structure, which restrained the flammable gases into the interior. Thus, the PAM/MsCa aerogel exhibited better flame retardancy and smoke suppression than the PAM aerogel. The relative compositions of C and Ca elements in the PAM/KCa residue were 8.3 wt% and 19.5 wt%, respectively. The possible flame retardancy mechanism of the PAM/KCa aerogels was speculated (Figure 7b), and more inert gases (such as NH_3_) were released from the cross-linked PAM/KCa aerogels than those of PAM/MsCa aerogel, and the denser residue in the PAM/KCa aerogel was also more beneficial to insulate heat transfer and the diffusion of the small molecule combustible gases. As a result, the flame retardancy of the PAM/KCa aerogel is better than that of the PAM/MsCa aerogel.

## 4. Conclusions

A series of highly efficient flame-retardant PAM composite aerogels were fabricated. After the cross-linking of silanes, the cell densities of the PAM/Ms and PAM/K aerogels increased, and the compressive moduli was obviously enhanced. However, the flame retardancy of the PAM/Ms and PAM/K slightly decreased. The compressive moduli of the PAM/MsCa and PAM/KCa aerogels dramatically increased through interpenetrating cross-linking network, approximately 10.5- and 8.2-fold of that of PAM aerogel, respectively. Meanwhile, the LOI values of the PAM/MsCa and PAM/KCa aerogels greatly increased to 40.5% and 56.8%, respectively. The cone calorimeter result indicates that the flame retardancy of PAM/MsCa and PAM/KCa composite aerogels obviously improved, and the smoke toxicity was greatly suppressed. The high flame-retardant PAM composite aerogels are promising in the flame-retardant material fields as an alternative to petroleum-based foams.

## Figures and Tables

**Table 1:** The physical and mechanical properties of different PAM composite aerogels.

Sample	Density /g·cm^−3^	Compressive Modulus /MPa	Specific Modulus /m^2^·s^−2^	Porosity /%
PAM	0.106 ± 0.001	2.1 ± 0.4	19.5	99.2
PAM/Ms	0.130 ± 0.005	5.3 ± 0.4	40.9	98.5
PAM/MsCa	0.273 ± 0.004	17.4 ± 1.1	63.7	95.9
PAM/K	0.156 ± 0.002	5.7 ± 0.1	36.3	98.2
PAM/KCa	0.284 ± 0.003	22.1 ± 0.4	77.8	96.8

**Table 2:** Thermal properties data of the different PAM composite aerogels (N_2_ atmosphere).

Sample	*T*_5%_ (°C)	*T_max1_* (°C)	*T_max2_* (°C)	*dW/dT* (%·min^−1^)	Char Yield (%)
PAM	242.3	290.9	/	6.3	52.9
PAM/Ms	234.0	301.9	428.7	7.7	51.5
PAM/MsCa	157.2	273.8	448.4	1.8	64.1
PAM/K	230.5	242.1	445.2	3.5	46.2
PAM/KCa	171.9	265.3	456.9	2.9	49.6

**Table 3:** LOI and UL-94 rating of the different PAM composite aerogels.

Sample	LOI (%)	t_1_/t_2_ (s/s)	UL-94 Rating
PAM	27.0	6/1	V-0
PAM/Ms	26.8	9/1	V-0
PAM/MsCa	40.5	3/0	V-0
PAM/K	26.5	8/1	V-0
PAM/KCa	56.8	2/0	V-0

**Table 4:** Cone calorimeter test data of different PAM composite aerogels at a heat flux of 35 kW/m^2^.

Sample	TTI (s)	pHRR (kW·m^−2^)	THR (MJ·m^−2^)	TSR (m^2^·m^−2^)	FPI (s·m^2^·kW^−1^)	THR/TML (MJ·m^−2^·g^−1^)	CR (%)
PAM	6	94.0	10.3	21.6	0.06	1.61	50.0
PAM/Ms	7	114.8	10.9	41.1	0.06	1.88	47.7
PAM/MsCa	44	57.4	3.2	8.6	0.77	0.51	69.0
PAM/K	9	112.2	12.7	64.5	0.08	1.93	46.8
PAM/KCa	58	62.5	2.7	5.5	0.93	0.46	69.6

## Data Availability

Not applicable.

## References

[1] Zheng Q.F., Xie R.S., Fang L.M., Cai Z.Y. (2018). Oxygen-deficient and nitrogen-doped MnO2 nanowire-reduced graphene oxide-cellulose nanofibrilaerogel electrodes for high-performance asymmetric supercapacitors. J. Mater. Chem. A.

[2] Yu M., Han Y.Y., Li J. (2018). Three-dimensional porous carbon aerogels from sodium carboxymethyl cellulose/poly(vinyl alcohol) composite for high-performance supercapacitors. J. Porous Mat..

[3] Wang Z.C., Wei R.B., Gu J.W. (2018). Ultralight, highly compressible and fire-retardant graphene aerogel with self-adjustable electromagnetic wave absorption. Carbon.

[4] Liu Y.N., Su Y.L., Guan J.Y. (2018). Asymmetric aerogel membranes with ultrafast water permeation for the separation of oil-in-water emulsion. ACS Appl. Mater. Interface.

[5] Chaudhary J.P., Vadodariya N., Nataraj S.K., Meena R. (2015). Chitosan-based aerogel membrane for robust oil-in-water emulsion separation. ACS Appl. Mater. Interface.

[6] She J., Tian C., Wu Y., Li X., Luo S., Qing Y., Jiang Z. (2018). Cellulose Nanofibrils Aerogel Cross-Linked by Poly(vinyl alcohol) and Acrylic Acid for Efficient and Recycled Adsorption with Heavy Metal Ions. J. Nanosci. Nanotechnol..

[7] Jiang J.X., Zhang Q.H., Zhan X.L., Chen F.Q. (2017). Renewable, biomass-derived, honeycomblike aerogel as a robust oil absorbent with two-way reusability. ACS Sustain. Chem. Eng..

[8] Zheng Q.F., Cai Z.Y., Gong S.Q. (2014). Green synthesis of polyvinyl alcohol (PVA)-cellulose nanofibril (CNF) hybrid aerogels and their use as superabsorbents. J. Mater. Chem. A.

[9] Zhao Y.Q., Liang Y.H., Zhao X.Z., Jia Q.Y., Li H.S. (2011). Preparation and microstructure of CuO-CoO-MnO/SiO_2_ nano-composite aerogels and xerogels as catalyst carriers. Prog. Nat. Sci-Mater..

[10] Wu T., Chen M.X., Zhang L., Xu X.Y., Liu Y., Yan J., Wang W., Gao J.P. (2013). Three-dimensional graphene-based aerogels prepared by a self-assembly process and its excellent catalytic and absorbing performance. J. Mater. Chem. A.

[11] Lee J.K., Gould G.L., Rhine W. (2009). Polyurea based aerogel for a high performance thermal insulation material. J. Sol-Gel Sci. Technol..

[12] Wu X.D., Cui S., Wang L., Shao G.F., Jiao J., Shen X.D. (2015). Advance in research of high temperature resistant aerogel used as insulation material. Mater. Rev..

[13] Wiener M., Reichenauer G., Braxmeier S., Hemberger F., Ebert H.P. (2009). Carbon aerogel-based high-temperature thermal insulation. Int. J. Thermophys..

[14] Kargarzadeh H., Huang J., Lin N., Ahmad I., Mariano M., Dufresne A., Thomas S., Gałeski A. (2018). Recent developments in nanocellulose-based biodegradable polymers, thermoplastic polymers, and porous nanocomposites. Prog. Polym. Sci..

[15] Liu H.Z., Geng B.Y., Chen Y.F., Wang H.Y. (2017). Review on the aerogel-type oil sorbents derived from nanocellulose. ACS Sustain. Chem. Eng..

[16] Chen H.-B., Schiraldi D.A. (2018). Flammability of Polymer/Clay Aerogel Composites: An Overview. Polym. Rev..

[17] Liu A., Medina L., Berglund L.A. (2017). High-Strength Nanocomposite Aerogels of Ternary Composition: Poly(vinyl alcohol), Clay, and Cellulose Nanofibrils. ACS Appl. Mater. Interfaces.

[18] Chen W.S., Yu H.P., Li Q., Liu Y.X., Li J. (2011). Ultralight and highly flexible aerogels with long cellulose I nanofibers. Soft Matter.

[19] Yang L., Mukhopadhyay A., Jiao Y.C., Yong Q., Chen L., Xing Y.J., Hamel J., Zhu H.L. (2017). Ultralight, highly thermal insulating and fire resistant aerogel by encapsulating cellulose nanofiber with two-dimensional MoS_2_. Nanoscale.

[20] Zheng Q., Javadi A., Sabo R., Cai Z.Y., Gong S.Q. (2013). Polyvinyl alcohol (PVA)–cellulose nanofibril (CNF)–multiwalled carbon nanotube (MWCNT) hybrid organic aerogels with superior mechanical properties. RSC Adv..

[21] Zhang X., Ziemer K.S., Zhang K., Ramirez D., Li L., Wang S., Hope-Weeks K.J., Weeks B.L. (2015). Large-area preparation of high-quality and uniform three-dimensional graphene networks through thermal degradation of graphene oxide–nitrocellulose composites. ACS Appl. Mater. Interface.

[22] Korhonen J.T., Kettunen M., Ras R.H., Ikkala O. (2011). Hydrophobic nanocellulose aerogels as floating, sustainable, reusable, and recyclable oil absorbents. ACS Appl. Mater. Interface.

[23] Chen H.B., Shen P., Chen M., Zhao H.B., Schiraldi D.A. (2016). Highly efficient flame retardant polyurethane foam with alginate/clay aerogel coating. ACS Appl. Mater. Interface.

[24] Li Y., Zhao M., Chen J., Fan S., Liang J., Ding L., Chen S. (2016). Flexible chitosan/carbon nanotubes aerogel, a robust matrix for in-situ growth and non-enzymatic biosensing applications. Sens. Actuators B Chem..

[25] Chen H.B., Chiou B.S., Wang Y.Z., Schiraldi D.A. (2013). Biodegradable pectin/clay aerogels. ACS Appl. Mater. Interface.

[26] Zuo L.Z., Fan W., Zhang Y.F., Zhang L.S., Gao W., Huang Y.P., Liu T.X. (2017). Graphene/montmorillonite hybrid synergistically reinforced polyimide composite aerogels with enhanced flame-retardant performance. Compos. Sci. Technol..

[27] Zhang Q.Q., Hao M.L., Xu X., Xiong G.P., Li H., Fisher T.S. (2017). Flyweight 3D graphene scaffolds with microinterface barrier-derived tunable thermal insulation and flame retardancy. ACS Appl. Mater. Interface.

[28] Wang L., Wu S.H., Dong X.Y., Wang R., Zhang L.Q., Wang J.L., Zhong J., Wu L.X., Wang X. (2018). A pre-constructed graphene–ammonium polyphosphate aerogel (GAPPA) for efficiently enhancing the mechanical and fire-safety performances of polymers. J. Mater. Chem. A.

[29] Guo W.W., Liu J.J., Zhang P., Song L., Wang X., Hu Y. (2018). Multi-functional hydroxyapatite/polyvinyl alcohol composite aerogels with self-cleaning, superior fire resistance and low thermal conductivity. Compos. Sci. Technol..

[30] Farooq M., Sipponen M.H., Seppälä A., Österberg M. (2018). Eco-friendly flame-retardant cellulose nanofibril aerogels by incorporating sodium bicarbonate. ACS Appl. Mater. Interface.

[31] Han Y.Y., Zhang X.X., Wu X.D., Lu C.H. (2015). Flame retardant, heat insulating cellulose aerogels from waste cotton fabrics by in situ formation of magnesium hydroxide nanoparticles in cellulose gel nanostructures. ACS Sustain. Chem. Eng..

[32] Yang Z.Y., Li H.K., Niu G., Wang J., Zhu D.C. (2021). Poly(vinylalcohol)/chitosan-based high-strength, fire-retardant and smoke-suppressant composite aerogels incorporating aluminum species via freeze drying. Compos. Part B.

[33] Trochimczuk A.W., Kabay N., Arda M., Streat M. (2004). Stabilization of solvent impregnated resins (SIRs) by coating with water soluble polymers and chemical crosslinking. React. Funct. Polym..

[34] Shang K., Ye D.D., Kang A.H., Wang Y.T., Liao W., Xu S., Wang Y.Z. (2017). Robust and fire retardant borate-crosslinked poly (vinyl alcohol)/montmorillonite aerogel via melt-crosslink. Polymer.

[35] Wu N.J., Niu F.K., Lang W.C., Xia M.F. (2019). Highly efficient flame-retardant and low-smoke-toxicity poly(vinyl alcohol)/alginate/montmorillonite composite aerogels by two-step crosslinking strategy. Carbohyd. Polym..

[36] Wang Y.T., Zhao H.B., Degracia K., Han L.X., Sun H., Sun M.Z., Wang Y.Z., Schiraldi D.A. (2017). Green approach to improving the strength and flame retardancy of poly(vinyl alcohol)/clay aerogels: Incorporating biobased gelatin. ACS Appl. Mater. Interface.

[37] Madyan O.A., Fan M. (2019). Organic functionalization of clay aerogel and its composites through in-situ crosslinking. Appl. Clay Sci..

[38] Si Y., Wang X.Q., Dou L., Yu J.Y., Ding B. (2018). Ultralight and fire resistant ceramic nanofibrous aerogels with temperature-invariant superelasticity. Sci. Adv..

[39] Wu N.J., Li X.T. (2014). Flame retardancy and synergistic flame retardant mechanisms of acrylonitrile-butadiene-styrene composites based on aluminum hypophosphite. Polym. Degrad. Stab..

[40] Wu N.J., Lang S.G. (2016). Flame retardancy and toughness modification of flame retardant polycarbonate/acrylonitrile-butadiene-styrene/AHP composites. Polym. Degrad. Stab..

